# The expanding boundaries of sphingolipid lysosomal storage diseases; insights from Niemann–Pick disease type C

**DOI:** 10.1042/BST20220711

**Published:** 2023-10-16

**Authors:** Frances M. Platt

**Affiliations:** Department of Pharmacology, University of Oxford, Mansfield Road, Oxford OX1 3QT, U.K.

**Keywords:** inborn errors of metabolism, lysosomal storage diseases, lysosomes, mycobacteria, sphingolipids, Tangier disease

## Abstract

Lysosomal storage diseases are inborn errors of metabolism that arise due to loss of function mutations in genes encoding lysosomal enzymes, protein co-factors or lysosomal membrane proteins. As a consequence of the genetic defect, lysosomal function is impaired and substrates build up in the lysosome leading to ‘storage’. A sub group of these disorders are the sphingolipidoses in which sphingolipids accumulate in the lysosome. In this review, I will discuss how the study of these rare lysosomal disorders reveals unanticipated links to other rare and common human diseases using Niemann–Pick disease type C as an example.

## Introduction

Lysosomal storage diseases (LSDs) are a group of rare inborn errors of metabolism [1]. At the cellular level, they are characterised by the accumulation (‘storage') of macromolecular substrates in the lysosome [1]. Inherited mutations in genes encoding lysosomal enzymes account for the majority of these diseases, with other lysosomal disorders arising from defects in genes encoding lysosomal membrane proteins or proteins that affect lysosomal function through other mechanisms [1]. Many of these diseases are neurodegenerative and all are life limiting. Clinical heterogeneity is common and in part reflects how much residual function is retained by the mutant protein [1].

The majority of LSDs are inherited as autosomal recessive traits and many patients are compound heterozygotes, with the two alleles of the affected gene carrying different mutations. Predicting the impact of two different mutations in combination is challenging, particularly as there are genetic, epigenetic and environmental modifying factors that in combination with the inherited mutation(s) lead to a broad spectrum of clinical presentations characteristic of these diseases [1].

The macromolecules stored in these disorders are biochemically diverse and include glycoproteins, lipids, glycogen, nucleic acids and mucopolysaccharides [1]. Within the broad class of lipid storage diseases, a subgroup involves storage of sphingolipids and are collectively termed the sphingolipidoses. Sphingolipids have a sphingoid backbone to which a fatty acid of variable chain length can be attached to form ceramide. Ceramide can be further modified, for example by the addition of carbohydrate head groups to generate glycosphingolipids (GSLs). Sphingosine is generated during sphingolipid catabolism and was first discovered by Johann Ludwig Thudichum (1829–1901) in 1874 when he was fractionating ethanolic brain extracts. He isolated galactosylceramide and following acid hydrolysis generated galactose, a fatty acid and a third molecule with an ‘alkaloidal nature' and enigmatic properties that he termed sphingosine. He therefore invoked Greek mythology using the riddle of the Sphinx as his inspiration for naming this class of lipids [2].

Thudichum's findings were published in 1884 in his seminal work entitled ‘A treatise on the chemical constitution of the brain'. An original copy is held by the Royal College of Physicians in London.

**Figure 1.**
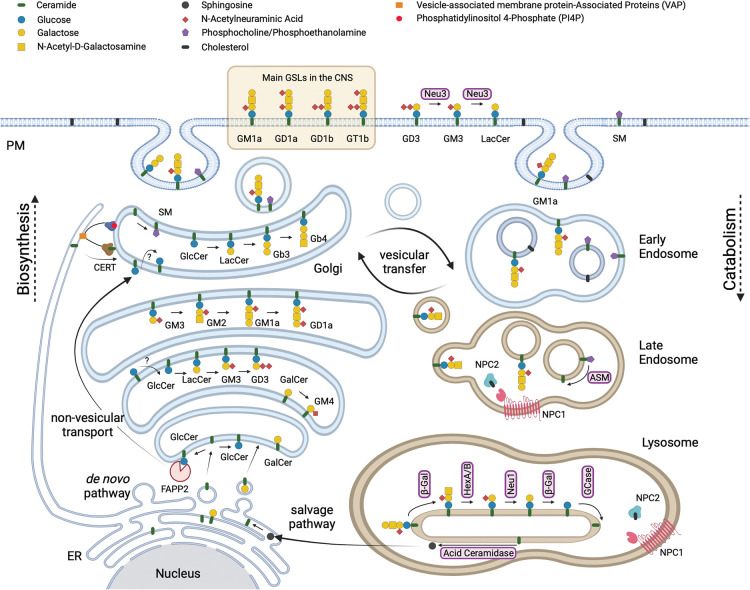
Schematic representation of the biosynthesis of sphingolipids in the ER and Golgi and their catabolism in the lysosome. This figure was adapted from Wallom et al. [64] and created using BioRender.com.

**Figure 2.**
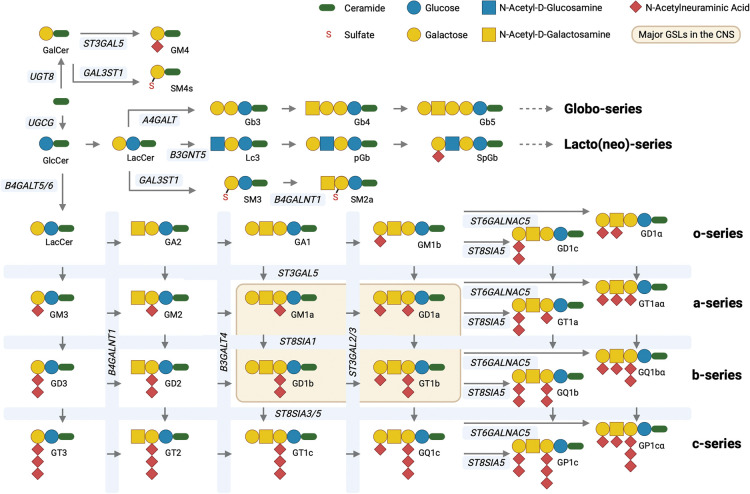
Schematic representation of GSL biosynthesis highlighting the key enzymes involved and the resulting GSL series and structures generated. The figure was created using BioRender.com.

The enigma of the biological functions of sphingolipids persisted for another 100 years before the roles of this complex class of lipids began to be elucidated [3]. Many of them are signalling lipids including ceramide, sphingosine and sphingosine-1-phosphate [3,4]. To this day, their functions remain incompletely understood and they are experimentally difficult to manipulate. This is because they are the product of complex, multi-enzyme biosynthetic pathways (Figure 1) that starts in the endoplasmic reticulum (ER) with the biosynthesis of ceramide through the action of multiple ceramide synthases that confer the differential acyl chain lengths of ceramide [5]. Ceramide is then transported to the Golgi apparatus where the glycosylation of ceramide can occur [6–8] and for the majority of GSLs begins with the transfer of glucose to ceramide on the external leaflet of an early Golgi compartment. Glucosylceramide is then flipped into the Golgi lumen and is further modified by sequentially acting transferases to form a diverse family of neutral and charged GSLs, including sialic acid containing gangliosides that are the major GSLs of the brain [9,10] (Figure 2). The transfer of galactose to ceramide in the lumen of the ER occurs to form GalCer that can be modified in the Golgi to form sulphatide and GM4, all of which are abundant GSLs in myelin (Figure 2).

As part of membrane turn over, sphingolipids are routed to the lysosome and degraded by a catabolic pathway that involves not only lysosomal hydrolases but also some protein co-factors that act as ‘liftases' to allow soluble hydrolases to access the cleavage site on the hydrophobic membrane embedded sphingolipid [11,12]. Although these pathways can be experimentally manipulated by knocking out genes encoding key enzymes in the pathway or pharmacologically inhibiting biosynthetic enzymes, precursors build-up in addition to losing downstream products of this complex pathway. Therefore, interpreting the biological consequences of manipulating sphingolipid biosynthesis and linking them to a specific lipid species is particularly challenging.

Insights into the functions of sphingolipids have therefore also been derived from the study of rare inherited metabolic diseases in which sphingolipid pathways are affected [11]. By understanding the disease state, insights into normal function can often be gleaned in a way that would be difficult through standard experimental approaches. To date two ganglioside biosynthetic diseases have been identified involving autosomal inherited mutations in GM2 and GM3 synthase [13,14]. GM3 synthase deficiency presents as a severe epilepsy where as GM2 synthase deficiency is a spastic paraplegia.

**Figure 3.**
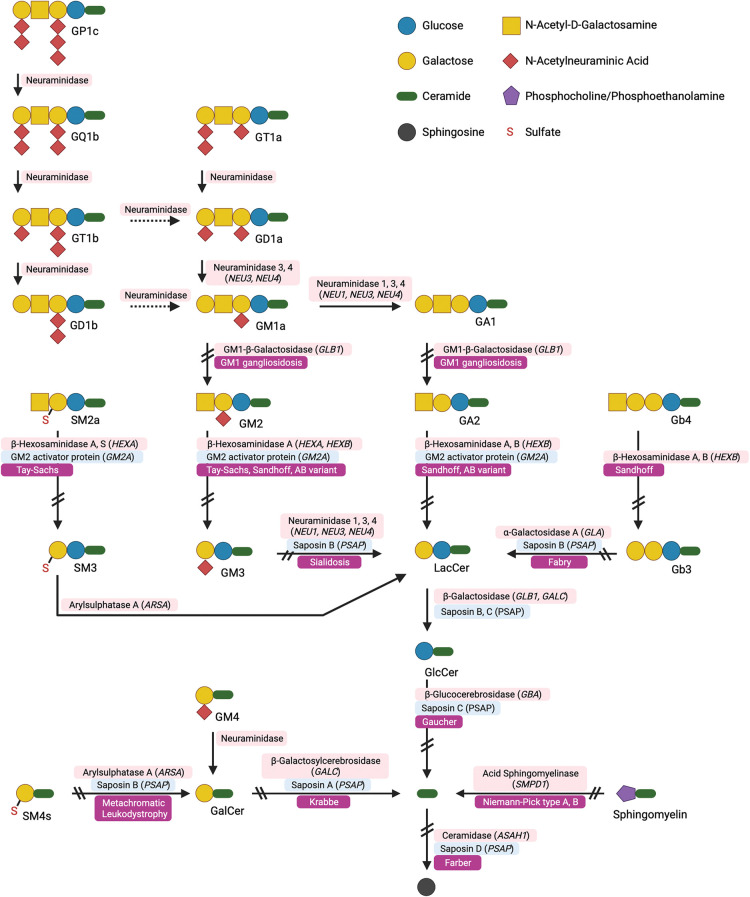
Schematic representation of GSL catabolism highlighting the lysosomal storage diseases arising from defects in lysosomal enzymes. This figure was adapted from Breiden and Sandhoff [65] and created using BioRender.com.

A major class of inborn errors of sphingolipid metabolism are the LSDs [1]. The primary sphingolipidoses arise most typically from autosomal recessive mutations in catabolic lysosomal enzymes or their co-factors (Figure 3). However, secondary sphingolipidoses also occur in which the primary defect is not directly involved in sphingolipid metabolism but sphingolipids are stored through other mechanisms [15].

In this article, I will focus on one specific secondary sphingolipid LSD, Niemann–Pick disease type C (NPC), and illustrate how complex and interwoven this single disease is with other rare disorders, but also surprisingly with much more common diseases. The insights gained from studying NPC are therefore informing not only our basic understanding of lysosomal lipid trafficking and lysosomal function, but also some unanticipated ways of understanding and potentially treating this group of disparate, but mechanistically linked diseases. This disease also highlights how lysosomal sphingolipid storage extends beyond the classical LSDs, extending disease classification boundaries in some unexpected ways.

## Niemann–Pick disease type C

Niemann–Pick type C is a progressive neurodegenerative LSD that involves lipid storage in the central nervous system and peripheral organs [16]. NPC typically presents in infancy or childhood [1,17] but adult onset cases also occur and are likely underdiagnosed [18]. NPC occurs at a frequency of 1: 100 000 live births and is inherited as an autosomal recessive trait [18].

NPC can be caused by mutations in two independent genes, *NPC1* or *NPC2* [16]. Neither of the lysosomal proteins (NPC1 and NPC2) encoded by these two genes function as lysosomal enzymes, but instead work cooperatively in an enigmatic pathway, often referred to as the ‘NPC pathway'. Another unusual feature of NPC is that it results in a very complex pattern of lipid storage, including LDL-derived cholesterol, sphingoid bases (e.g. sphingosine and sphinganine) and all sphingolipids including glycosphingolipids (GSLs) and sphingomyelin [16]. At the cellular level, fusion between late endosomes and lysosomes is impaired and LDL-derived cholesterol fails to efficiently reach the ER leading to downstream consequences, for example on cholesterol regulation [19] and the P450 xenobiotic/drug metabolism system [20].

**Figure 4.**
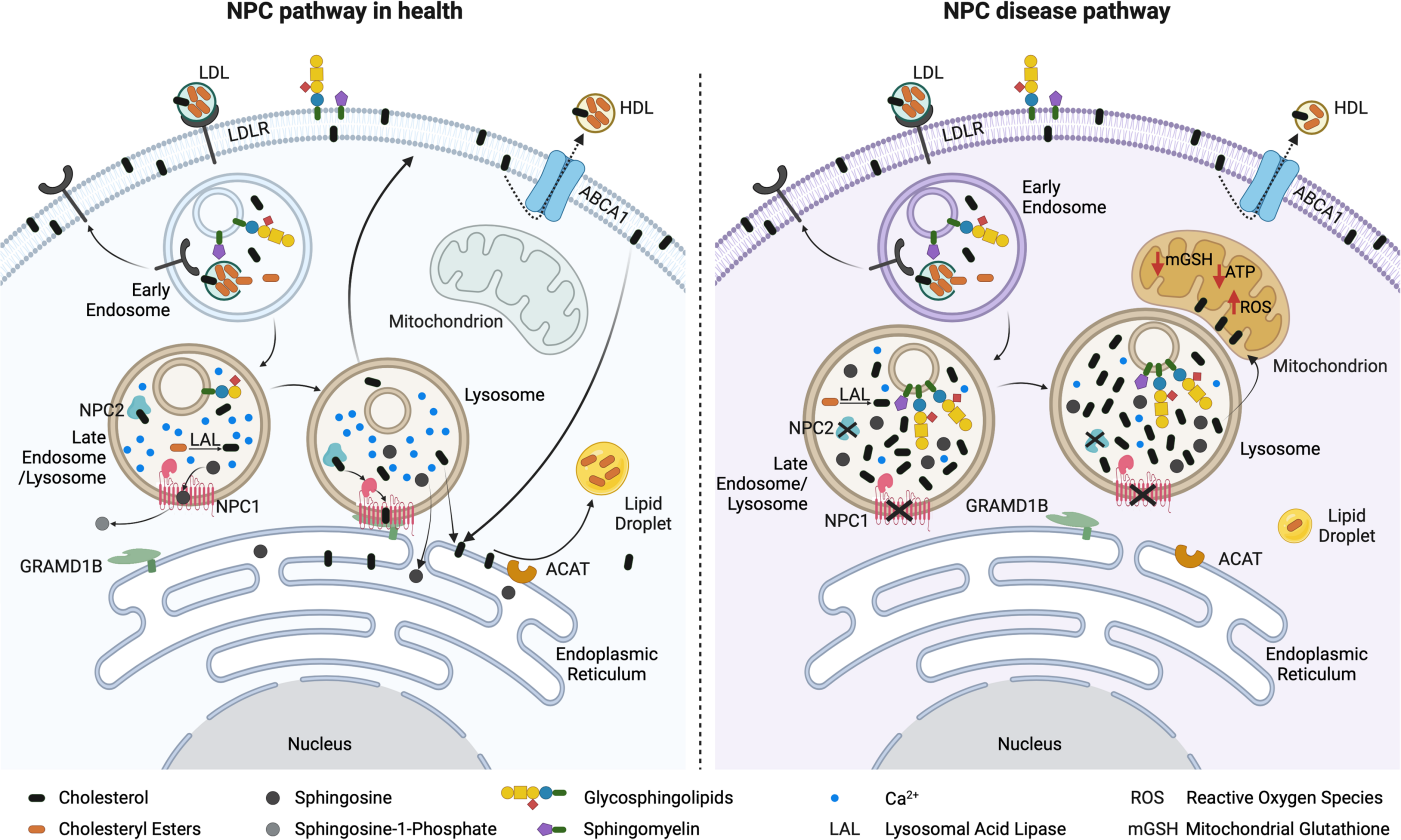
Schematic representation of the NPC lipid trafficking pathway in healthy cells and NPC deficient cells. The figure was created using BioRender.com.

LDL-derived cholesterol has a complex itinerary within eukaryotic cells [21]. This includes a lysosome to ER route that is via the plasma membrane and is dependent on phosphatidylserine [22] (Figure 4). In addition, there is a direct lysosome to ER pathway in which NPC1 plays a key role as part of a lysosome: ER tethering complex [23] (Figure 4). Specifically, NPC1 on the lysosome limiting membrane forms a tether with the ER localised lipid transfer protein Gramd1b (Figure 4). Cholesterol and potentially other lipids move at these inter-organelles contact sites [23,24]. Furthermore, there is a Ca^2+^ signalling defect in NPC cells that specifically affects acidic Ca^2+^ stores, including lysosomes and lysosome related organelles [25–27]. Lysosomes in NPC deficient cells have reduced levels of Ca^2+^, likely due to a defect in store filling that also occurs at lysosome: ER contact sites [28,29].

NPC1 is a member of the RND permease family of transporters [30] that evolved in prokaryotes and efflux multiple substrates from bacteria allowing them to live in chemically hostile environments [31]. Mammals express a limited number of RND permease family members including NPC1 and the developmental protein Patched [31,32]. Yeast express an orthologue of NPC1 termed Ncr1 and this ancestral protein that resides in the yeast vacuole can rescue mammalian NPC1 deficient cells, consistent with a conserved function of this protein in the lysosome over evolutionary time [33].

In contrast with NPC1, NPC2 is a small, soluble, globular protein that is targeted to the lysosome via the mannose-6-phosphate pathway and has a binding cavity that accommodates free cholesterol [34]. This NPC2 bound cholesterol is transferred to the NPC1 protein that resides in the limiting lysosomal membrane [35], where its binding to the sterol sensing domain induces an ordered structure of NPC1 [36]. These findings are consistent with NPC1 being a cholesterol-regulated protein that is important for membrane contact site formation at the ER and the efflux of lipids by the action of the lipid transfer protein Gramd1b [23,37]. Recent studies have demonstrated that sphingosine directly interacts with the NPC1 protein [38] supporting a potential sphingosine transport role for NPC1 as previously suggested based on sphingosine being the first metabolite to be stored following inhibition of NPC1 and is upstream of the lysosomal Ca^2+^ defect [38,39].

## The therapeutic landscape

The complexity of NPC offers many different routes to potentially treat this rare monogenic disease including gene therapy [40], small molecules targeting cholesterol storage (cyclodextrin) [41], small molecule substrate reduction therapy to reduce GSL storage, (miglustat) [42], small molecule enhancement of the heat shock protein response (arimoclomol) [43] and small molecule metabolic enhancement therapeutic (acetyl-l-leucine) [44] (Table 1). Indeed, these therapies have demonstrated efficacy in animal models, progressed to clinical trials and in one case (miglustat), achieved regulatory approval (Table 1) [45].

**Table 1:** Summary of NPC therapeutics and their differential stages of development

Therapeutic	Mechanism of action	Efficacy in animal models	Clinical trials	Approval
Miglustat	SRT	+	+	2009
Cyclodextrin	Induces exocytosis	+	+	-
Arimoclomol	HSP70 inducer	+	+	-
Acetyl-L-leucine	Metabolic enhancer	+	+	-
NPC1 gene therapy	Gene Therapy	+	-	-

For more information see Platt et al. [1]. The table was created using BioRender.com.

Miglustat was approved for the treatment of NPC in 2009 by the EMA and is a substrate reduction therapy drug that inhibits the Golgi enzyme glucosylceramide synthase, reducing levels of GSLs so fewer GSLs require catabolism in the lysosome [46]. It is not completely clear if this is the only mechanism of action of miglustat explaining its efficacy in NPC as inhibition of the non-lysosomal glucocerebrosidase GBA2 has also been implicated [47]. Miglustat also has the ability to reduce fibrosis and it is possible that this may also contribute to miglustat's efficacy in individuals with NPC [34]. Irrespective of its precise mechanisms of action, analysis of 10 years of experience with miglustat treatment of NPC patients shows this CNS penetrant drug slows disease progression [48]. Miglustat extends the life span of affected individuals by approximately a decade, compared with natural history data from untreated patients [48].

## NPC and Tangier disease

Although the majority of our understanding of NPC has come from basic research, a clinical case treated with the standard of care drug miglustat provided an unexpected insight into how NPC is linked to an apparently unrelated ultra-rare metabolic disease.

A patient misdiagnosed with NPC was prescribed miglustat and responded well to treatment [49]. When this individual was subsequently correctly diagnosed they were found to have Tangier disease (familial alpha-lipoprotein deficiency), an ultra-rare inherited metabolic disorder that arises from mutations in *ABCA1* [50]. ABCA1 is a prototypic ABC transporter that plays a key role in reverse cholesterol transport out of cells and is the rate-limiting step in the initial formation of HDL apolipoprotein particles [51,52]. ABCA1 is expressed in most tissues, particularly in tissue macrophages, with highest expression in the liver and lung. It remains unclear if the action of ABCA1 is confined to the cell surface as ABCA1 is also found in late endosomes and lysosomes [52,53]. It had previously been shown that levels of NPC1 increase in Tangier disease cells, potentially in an attempt to rebalance cholesterol transport pathways when ABCA1 is deficient [54]. Furthermore, inducing expression of ABCA1 using activators such as LXR in NPC cells rescues cellular phenotypes of NPC, but in an NPC2 dependent manner [55]. Also, individuals with NPC have low circulating HDL levels as a secondary consequence of reduced ABCA1 levels.

A central question arising from the misdiagnosed clinical case [49] is why would a patient with a mutation in *ABCA1* respond to a GSL substrate reduction therapy drug (miglustat) that is approved for treating NPC patients? To investigate this further we profiled the cell biological changes known to occur in NPC patient cells and compared them with fibroblasts from Tangier disease patients [56]. We found that the two diseases phenocopied each other both biochemically (pattern of LDL-derived cholesterol and sphingolipids stored) and in terms of the lysosomal calcium defect (reduced acidic store Ca^2+^ levels) [56]. Furthermore, miglustat treatment of Tangier cells *in vitro* rescued these cellular phenotypes [56]. Although the precise mechanism leading to the secondary induction of NPC cellular phenotypes in Tangier disease cells remains to be determined, this unexpected finding reinforces the links between NPC1 and ABCA1 function [57]. Currently, a novel single Tangier case clinical trial (*n* = 1 trial cycling on and off miglustat treatment) is being conducted in the UK (EudraCT/CTIS number2020-005505-13). If successful it is anticipated that this will enable other Tangier disease patients to be prescribed miglustat, as its ultra-rare status makes Tangier disease a truly Orphan disorder in terms of Pharma interest. However, drug repurposing for this disease is both practical and realistic.

A clear implication of this convergence between NPC and Tangier is that Tangier disease is a secondary LSD, adding another rare disease to the list of LSDs involving sphingolipid storage.

Another interesting observation linking ABC transporters to NPC came to light from a yeast screen studying the interacting partners of the yeast orthologue of NPC1, Ncr1 on the yeast vacuole (the equivalent of the mammalian lysosome) [24]. Ncr1 functions as part of a complex of which an ABC transporter Ycf1 is a partner [24]. Ycf1 is an orthologue of mammalian ABCC3 that is involved in multidrug resistance. This suggests that NPC1 and ABCC3 may work in concert to move lipids and other substrates from the inner leaflet of the lysosomal membranes to the outer leaflet to facilitate their egress at membrane contact sites. In plants, ABCC3 is induced by heavy metals such as cadmium facilitating their egress from the plant vacuole [58]. It is therefore conceivable that NPC1 and ABCC3 work in concert in the mammalian lysosome, facilitating the movement of a diverse range of substrates out of the late endocytic/lysosomal system at membrane contact sites.

## NPC and infectious diseases

An unexpected link between NPC and a very common infectious disease was made in 2016 [59]. *Mycobacterium tuberculosis* (*Mtb*) has the unusual ability to infect and persist within host macrophages by subverting the host cell to prevent the fusion of the lysosome with the phagosome. How *Mtb* prevents phagosome: lysosome fusion remains incompletely understood [60]. Cells infected with *Mtb* have been known for many decades to induce a characteristic ‘foamy' macrophages as they accumulate free cholesterol [61]. Indeed, very unusually cholesterol is catabolised by *Mtb* and used as a carbon source facilitating mycobacterial survival [62]. Cholesterol ‘storage' is a shared feature with NPC, which could just be a superficial similarity or suggest a convergent mechanistic basis. Another shared cell biological feature of NPC and *Mtb* infection is that fusion between the lysosome and other organelles is impaired due to reduced levels of lysosomal Ca^2+^ [59]. For example, in NPC lysosomes don't fuse efficiently with late endosomes and autophagosomes. In the case of *Mtb* infection, lysosomes do not fuse with phagosomes [59].

A study was therefore conducted to determine whether these superficial similarities had a shared mechanistic basis [59]. It was found that all cellular phenotypes found in NPC cells occur in *Mtb* and *Mycobacterium bovis* (Bacillus Calmette–Guerin (BCG)) infected cells [59]. However, non-pathogenic mycobacteria such as *M. smegmatis* don't have these effects on host macrophages [59]. It was observed that when cells were infected with BCG it was not just the infected cells that exhibited NPC cellular phenotypes but also non-infected bystander cells [59]. This finding suggested a component of the pathogen was released from the mycobacterium and gained access to neighbouring uninfected cells. This mediator was found to be heat resistant and to reside in the lipid fraction of extracted cells [59]. When lipid extracts from pathogenic persistent mycobacteria were administered to healthy uninfected macrophages all NPC phenotypes were induced because the effector lipid(s) shed by the pathogen inhibited NPC1 function. These studies were then extended to non-tubercular pathogenic mycobacteria and they also were found to have lipids that inhibit NPC1 [59]. Furthermore, when *Mtb* was studied using lipid extracts from the major global families of *Mtb* they were all able to inhibit the NPC pathway suggesting that all modern era strains of this pathogen evolved from an ancestral strain that had this ability to block NPC1 function [63]. One extant strain that represents an ancestral lineage is *M. canettii* and when lipid extracts were tested, no inhibition of NPC1 and the NPC pathway were detected, suggesting that the ability to target NPC1 evolved after divergence of *Mtb* from a common ancestor shared with *M. canettii* [63].

**Figure 5.**
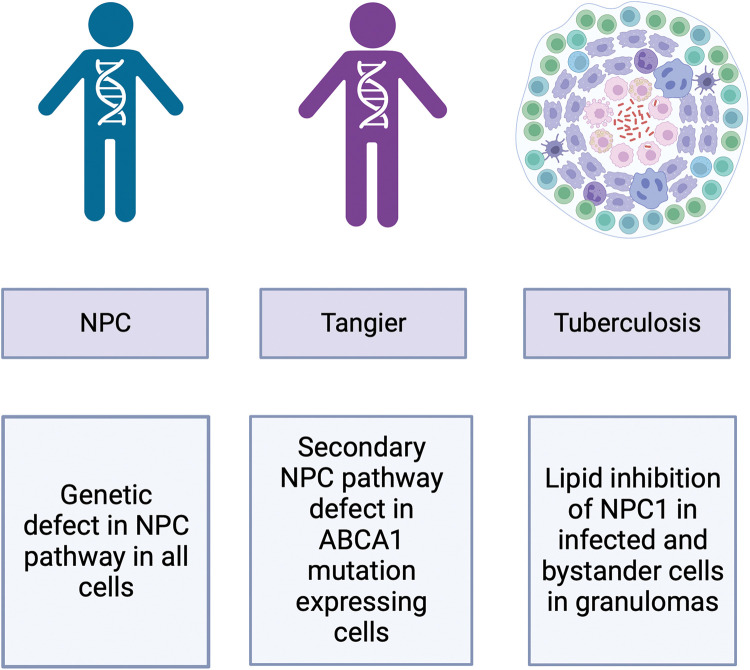
Schematic diagram summarising how NPC1 defects occur in different diseases. A global defect in the NPC pathway is present in all cells in the primary lysosomal storage disease Niemann–Pick disease type C, the NPC pathway defect secondary to ABCA1 deficiency is present in Tangier disease cells and the lipid mediated inhibition of NPC1 by the intracellular pathogen, *Mycobacterium tuberculosis* leads to storage in infected and bystander cells*.* The figure was created using BioRender.com.

These findings highlight a third category of LSDs in which certain cell types develop the LSD phenotype (infected cells and neighbouring uninfected cells) not systemically as in the primary disorders (Figure 5). In addition, these discoveries suggest that some therapies developed for treating NPC may have utility in promoting the clearance of intracellular pathogens. One drug that has shown utility in NPC is curcumin, a weak SERCA antagonist. This drug leads to transient elevation in cytosolic Ca^2+^ levels that compensate for the impaired Ca^2+^ release from lysosomes and has shown benefit in a mouse model of NPC [39]. We hypothesised that curcumin may facilitate fusion between lysosomes and phagosomes by elevating cytosolic Ca^2+^ and so we tested this in a zebra fish model infected with the fish pathogen *Mycobacterium marinum* [59]. After 24 h of exposure to the drug the mycobacterial burden in the fish was significantly reduced providing the first *in vivo* proof of principle that this host-targeted anti-microbial strategy merits further research to potentially progress it to clinical studies in the future.

In conclusion, from the study of NPC it has become clear that the NPC pathway is involved in many more diseases than previously appreciated, ranging from ultra-rare metabolic disorders such as Tangier disease through to very common infectious diseases including TB (Figure 5). Furthermore, the drugs developed to treat NPC may have unanticipated utility as host-targeted anti-microbial drugs. These studies also highlight the complexity of monogenic diseases and the role of sphingolipids in multiple aspects of cell biology that have only come to light through the study of rare LSDs.

## Perspectives

- Lysosomal storage diseases are a group of severe, often neurodegenerative metabolic disorders. One very active field of research focuses on sphingolipid storage diseases. By studying these diseases, we have learned a great deal about the function and regulation of sphingolipids and the lysosome. Despite their rarity, this knowledge has been used to innovate multiple approved therapies. In this review the primary focus is on the lysosomal storage disorder, NPC.
- A current focus in this field is on the mechanistic links between lysosomal sphingolipid storage disorders and other diseases. This has highlighted links to common neurodegenerative diseases such as Parkinson's and Alzheimer's [1], but also to infectious diseases [59]. This has demonstrated that many more diseases than we had previously appreciated are secondary LSDs, expanding the boundaries of how we classify these disorders.
- In the future, orphan disease therapies developed to treat LSDs will be trialled in mechanistically linked disparate diseases, expanding the benefit for patients.

## Open Access

Open access for this article was enabled by the participation of University of Oxford in an all-inclusive *Read & Publish* agreement with Portland Press and the Biochemical Society under a transformative agreement with JISC.

## Abbreviations

BCG: Bacillus Calmette–Guerin
ER: endoplasmic reticulum
GSLs: glycosphingolipids
LSDs: lysosomal storage diseases
NPC: Niemann–Pick disease type C
